# Characterisation of anaemia amongst school going adolescent girls in rural Haryana, India

**DOI:** 10.1017/S1368980022000210

**Published:** 2022-12

**Authors:** Aakriti Gupta, Harshpal Singh Sachdev, Umesh Kapil, Shyam Prakash, Ravindra Mohan Pandey, Hem Chandra Sati, Lokesh Kumar Sharma, Priti Rishi Lal

**Affiliations:** 1 Department of Food and Nutrition, Lady Irwin College, University of Delhi, New Delhi 110001, India; 2 Department of Pediatrics and Clinical Epidemiology, Sitaram Bhartia Institute of Science and Research, New Delhi, India; 3 Department of Human Nutrition, All India Institute of Medical Sciences, New Delhi, India; 4 Department of Laboratory Medicine, All India Institute of Medical Sciences, New Delhi, India; 5 Department of Biostatistics, All India Institute of Medical Sciences, New Delhi, India; 6 Department of Biochemistry, Dr. Ram Manohar Lohia Hospital, New Delhi, India

**Keywords:** Anaemia, Haemoglobin, Iron, Folate, Adolescent

## Abstract

**Objective:**

High burden of anaemia exists amongst rural adolescent girls in India. The objective of this study was to characterise anaemia in school going adolescent girls in rural Haryana, India.

**Design:**

Linear and multiple logistic regression analysis of data collected prior to an intervention trial was conducted. Participants were classified into anaemic (haemoglobin <12 g/dl) and non-anaemic group and were further classified into deficiencies of Fe, folate or vitamin B_12_, mixed, anaemia of other causes and inflammation.

**Setting:**

Three schools in Ballabgarh block of Faridabad District, Haryana, India.

**Participants:**

One hundered and ninety-eight non-anaemic and 202 anaemic adolescent girls (12–19 years).

**Results:**

Anaemic girls had 29·6 % Fe deficiency, 28·1 % folate or vitamin B_12_ deficiency, 15·8 % mixed deficiency and 9·7 % acute inflammation. Anaemia of other causes was found in 16·8 % of the anaemic participants. Girls with Fe and isolated folate deficiency had 2·5 times and four times higher odds of developing anaemia, respectively, as compared with non-anaemic girls. Fe deficiency with no anaemia was found amongst 11 % non-anaemic girls. Non-anaemic girls had a high prevalence of combined deficiency of folate or vitamin B_12_ (29·5 %) and acute inflammation (14·4 %).

**Conclusions:**

The current strategy of Fe and folic acid supplementation alone will not suffice for achieving the desired reduction in the prevalence of anaemia as unknown causes and anaemia of inflammation contribute to a substantial proportion of anaemia. Integrating other nutrition-specific components like improving water, sanitation and hygiene practices with the ongoing micronutrient supplementation program will comprehensively tackle anaemia. Unknown causes of anaemia warrant further research.

Anaemia in adolescent girls is a major public health problem in India with 40 % being afflicted^(1)^. Adolescent girls are vulnerable to anaemia due to regular loss of Fe through menstrual blood in addition to the overall accelerated increase in requirements for Fe due to rapid pubertal growth. Functional consequences of anaemia on growth and development occur even at mild levels or prior to onset of clinical stage of anaemia, making it the third leading cause of disability in the world^(2)^.

Some recent evidence challenges the earlier notion that Fe deficiency is the predominant contributor to anaemia globally^(3,4)^. Estimates suggest that less than half the cases of anaemia are due to Fe deficiency, and the other causes are unknown^(3–5)^. Anaemia due to inflammation has been reported to further lower the proportion of anaemia associated with Fe deficiency (20 % for pre-school children; 25 % for non-pregnant women of reproductive age)^(4)^. Most strategies in India focus on increasing Fe intake, despite the fact that anaemia is multifactorial in aetiology and its prevalence varies among population groups and in different areas and local conditions^(6)^. The National Nutritional Anemia Control Program (*Anemia Mukt Bharat*) provides weekly Fe and folic acid (IFA) supplementation (60 mg elemental Fe and 500 mcg of folic acid) and biannual de-worming to all in school children, through teachers and to out-of-school children, through quarterly Adolescent Health Day component of *Rashtriya Kishor Swasthya Karyakram* programme at *Anganwadi* centres^(7)^. However, a slow decline in the prevalence of anaemia has been observed amongst adolescent girls in India over the past decades as represented by National surveys^(8)^. With the current strategy and knowledge, we may not be able to achieve the national target of 3 % percentage points per annum reduction in the prevalence of anaemia among adolescent girls and women in the age group of 15–49 years.

Therefore, the present study was conducted to characterise anaemia, especially amongst rural adolescent girls as more than half of them reside in rural areas of the country^(9)^.

## Methods

### Study design, population and sampling techniques

The present study was nested within a community-based cluster-randomised controlled trial conducted to assess the impact of daily supplementation of IFA alone or in combination with vitamin B_12_ amongst adolescent girls in the age group of 12–19 years living in the rural Ballabgarh block of Faridabad District, Haryana, India. We have analysed and presented the cross-sectional data at baseline of 400 girls within the framework of the randomised control trial.

**Fig. 1:**
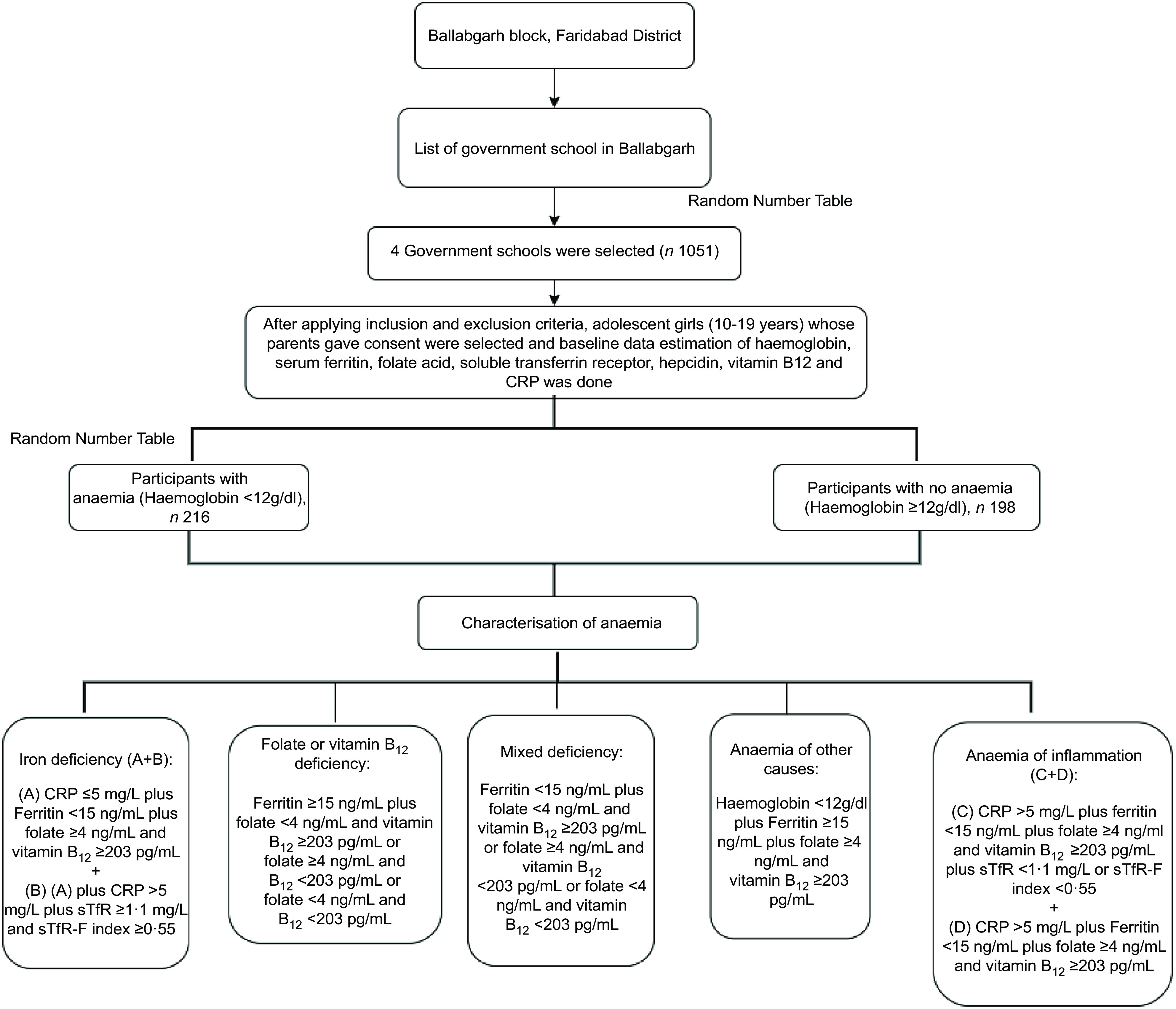
Summary of the recruitment process

Faridabad is predominantly (75·9 %) urban^(10)^ with 54·1 % of the adolescents being anaemic (haemoglobin <12 g/dl) as per NFHS-4^(11)^. We selected Ballabgarh block of Faridabad district as 98 % of its total population reside in rural areas^(10)^. According to the Annual Status of Education Report (2020), the overall school enrollment for rural adolescent girls has been above 90 % in India and around 93 % in the study area^(12)^. We compiled a list of all middle and senior secondary government schools in Ballabgarh block from which three schools in Dayalpur, Fatehpur Billoch and Chhainsa were selected for recruitment of study participants. All 1051 adolescent girls aged 12–19 years studying in 6–12th standard and who gave the informed consent were screened for haemoglobin. All non-anaemic participants with haemoglobin of >12 g/dl (*n* 198) were included in the study. Using random number table, 202 participants with mild anaemia (haemoglobin level of 10–11·9 g/dl: 41·7 %) and moderate anaemia (haemoglobin level of 7–9·9 g/dl: 51·9 %) were selected randomly. All fourteen participants (6·4 %) with severe anaemia (haemoglobin: <7 g/dl) were excluded from the study and were referred to the nearest primary health centre for treatment. The socio-demographic profile of enrolled and not enrolled participants in the present study from the original community-based cluster-randomised controlled trial has been presented in Table 1. A flow chart of participant selection is available in Fig. 1.

**Table 1:** Baseline measurements of socio-demographic profile of the enrolled and not enrolled participants

Baseline characteristics	Enrolled (*n* 400)		Not enrolled* (*n* 651)		*P* value
Age (years)	14·5	1·8	14·3	1·7	0·06
Per capita income (in Rs.)†					
p_50_	1250		1270		0·37
p_25_–p_75_	833–2000		800–2000		
Height (cm)	150·6	7·9	148·9	8·4	0·001
Weight (kg)	40·4	8·5	39·4	8·0	0·06
BMI (kg/m^2^)	13·3	2·5	13·1	2·3	0·27

* Not enrolled category includes those adolescent girls who were not selected from the community-based cluster-randomised controlled trial under which the present study was nested.

† Per capita income is expressed as the household’s monthly income divided by the number of family members, and values are expressed as p_50_ (p_25_–p_75_).

### Biochemical assessment

Haemoglobin estimation was undertaken using cyanmethhaemoglobin method^(13)^. Twenty microlitre of capillary blood was spotted on pre-labelled Whatman Filter paper no. 1. The filter paper was then allowed to dry and packed in zip pouch and transported to the central laboratory of All India Institute of Medical Sciences, New Delhi, India, for analysis. We used an indirect cyanmethhaemoglobin method using filter paper, as it was not feasible to transport whole venous blood within an hour under suitable conditions to the central laboratory.

Venous blood (5 ml) was drawn simultaneously in plain vacutainers (Beckton Dickinson, Franklin Lakes, NJ, USA) for the biochemical investigations of serum ferritin, soluble transferrin receptor (sTfR), hepcidin, folic acid and vitamin B_12_ utilising standard operating procedures. Blood samples were centrifuged within 2 h of collection. The serum samples were transported to the central laboratory for biochemical estimations and thereafter kept at −80 °C. Internal quality control was maintained using standards/controls with every batch of samples.

Serum ferritin was estimated by two-site sandwich immunoassay using direct chemiluminometric technique (Immulite-1000, Siemens, Berlin, Germany)^(14)^. Serum sTfR, hepcidin and folic acid levels were estimated by sandwich ELISA method^(15)^. Vitamin B_12_ estimation was done by competitive immunoassay using direct chemiluminescent technique (Immulite-1000, Siemens, Berlin, Germany)^(14)^. High-sensitivity C-reactive protein (CRP) level was estimated by solid phase ELISA technique^(15)^.

### Outcome: anaemia

The WHO cut-off (2011) of <12 g/dl for haemoglobin was utilised to define anaemia^(16)^. Participants were further classified as: mild (10–11·9 g/dl), moderate (7–9·9 g/dl) and severe (<7 g/dl)^(16)^.

### Exposures

Biochemical levels of Fe, sTfR, hepcidin, folic acid, vitamin B_12_ and CRP.

For the determination of Fe status, two biomarkers were used: ferritin and sTfR. Serum ferritin concentration of <15 ng/ml was utilised to reflect the storage Fe compartment. Serum ferritin concentration of <70 ng/ml was used as the cut-off for individuals with infection or inflammation (CRP > 5 mg/l)^(17)^. sTfR concentrations of >1·1 mg/l (ELISA kit-based cut-offs) were used to define functional Fe compartment and status of erythropoiesis (Kinesis DX, Cat No: K12-0282). Hepcidin concentration, a marker for assessing Fe homoeostasis, was defined using the ELISA kit-based cut-offs. The assay range of the kit was 50–800 ng/ml (Kinesis DX, Cat No: K12-1020). Elevated levels were defined as hepcidin >400 ng/ml and low levels as <100 ng/ml. Vitamin B_12_ deficiency was defined as serum vitamin B_12_ level <203 pg/ml and folate deficiency as a serum folate level <4 ng/ml^(18)^. Acute inflammation was defined as CRP > 5 mg/l^(19)^.

### Characterisation of anaemia

Participants were classified into the following aetiological categories^(5)^: (i) Fe deficiency was characterised as (A) ferritin deficiency plus sufficient folate and vitamin B_12_ levels amongst participants with CRP ≤ 5 mg/l; and (B) A plus elevated sTfR and sTfR–ferritin index in participants with CRP > 5 mg/l; (ii) folate or vitamin B_12_ deficiency was characterised as sufficient ferritin along with deficiency of either folate or vitamin B_12_ or both, irrespective of CRP concentrations; (iii) dimorphic anaemia/mixed deficiency was characterised as ferritin deficiency along with deficiency of either folate or vitamin B_12_ or both, irrespective of CRP concentrations; (iv) anaemia of other causes was characterised by low haemoglobin (<12 g/dl) with sufficient ferritin, folate and vitamin B_12_ level amongst participants with CRP ≤ 5 mg/l and (v) anaemia of inflammation was characterised amongst participants with CRP >5 mg/l as: (A) participants with ferritin deficiency along with sufficient folate, vitamin B_12_ and elevated sTfR or elevated sTfR–ferritin index and (B) sufficient ferritin, folate and vitamin B_12_ level.

### Potential confounders

Age, type of diet, wealth index, water source, consumption of deworming tablets (past 1 year), IFA tablets (past 30 d) and status of menstruation.

We used a pre-tested semi-structured questionnaire to elicit information on identification data, socio-demographic profile, type of house (*Kutcha* houses with mud walls and thatched/tiled roofs, semi-*pucca* houses with brick walls and tiled/asbestos/tin roofs and *pucca* houses with brick walls and reinforced cement concrete roofs), water source, toilet facility, diet and consumption of deworming tablets (past 1 year) and IFA tablets (past 30 d).

### Wealth index

We used a principal component analysis to construct a wealth assets index from the information collected on household ownership of consumer durables, the characteristics of the household’s dwelling and household landownership^(20)^.

### Anthropometry

Standing height was recorded barefoot, using a standard stadiometer (SECA 213) to the nearest 1 mm. Participants were weighed using a weighing scale (SECA 813) to the nearest 0·1 kg, wearing minimal clothing and without shoes. BMI was calculated as weight (kg)/height^2^ (m^2^). The WHO BMI *Z*-scores were used to classify adolescent girls as severe thinness, thinness, normal and overweight^(21)^. WHO height-for-age *Z*-scores classified children into normal, stunted and severely stunted categories^(21)^.

### Ethics statement

After describing the objectives and risks of the study, an informed consent was obtained from the parents/guardians of all the children. A written assent was obtained for children between 13 and 18 years. An additional oral assent was obtained from the children who were less than 13 years old in the presence of the parent/guardian representative. The consent was written for those parents/caretakers who are literate and oral (with a witness) along with thumb impression for those who were illiterate.

### Statistical analysis

Stata/se 13.0 version 13.0, StataCorp. software was used for statistical analysis of data. Quantitative data were expressed in mean & SD for normally distributed variables, median (25^th^ percentile, 75^th^ percentile) for non-normally distributed continuous variables and frequency (percentage, %) for categorical variables. We log-transformed serum ferritin to achieve normality and reported geometric mean and interquartile ranges. Two-sided *P*-value of <0·05 was regarded as statistically significant. We assessed association of different biochemical, socio-economic and demographic parameters with anaemia using Student’s *t*-test, Chi-square test or Fischer exact test. Linear regression was performed to assess the association of factors with haemoglobin level as the outcome variable. Multiple logistic regression was used to examine the factors associated with anaemia. We adjusted for age (in years), CRP (in mg/l), caste (scheduled caste and tribes (SC/ST)/Other Backward Classes (OBC) *v*. Others), source of drinking water (Handpump *v*. Others), reported consumption of IFA in past month (yes *v*. no) and menstruation status (no *v*. yes) in our analysis.

## Results

The general characteristics of the enrolled participants by anaemia status are presented in Table 1. Approximately 2/3^rd^ of the participants were thin with 33·5 % being severely thin (BMI < -3 SD). Stunting as defined by height for age <-2 SD was found in 18·2 % of the participants^(21)^.

### Comparison of participants with and without anaemia

Adolescents with anaemia were significantly different from those without anaemia in characteristics, namely, greater age (0·4 years, *P* = 0·022), SC/ST (57·9 % anaemic *v*. 43·3 % non-anaemic; *P* = 0·003), nuclear families (54·3 % anaemic *v*. 45·7 % non-anaemic; *P* = 0·029), protected drinking water supply (piped water) (45·4 % anaemic *v*. 54·5 % non-anaemic; *P* = 0·001), reported consumption of IFA tablets in the past month (48·2 % anaemic *v*. 51·8 % non-anaemic; *P* = 0·020) and menstruation (56·2 % anaemic *v*. 43·8 % non-anaemic; *P* < 0·000). The two groups were comparable for the other evaluated characteristics including height, weight, BMI, religion, per capita income, wealth index, type of house, type of toilet facility, type of diet and reported consumption of deworming tablets in the past month (Table 2).

**Table 2:** Characteristics of adolescent girls aged 12–19 years enrolled in the study, stratified by anaemia status

Parameters	Non-anaemia group (*n* 198)		Anaemia group (*n* 202)		*P* value
	*n*	%	*n*	%	
Age					
Mean	14·3		14·7		0·022
SD	1·8*		1·9*		
Age category					
12–15 years	149	53·4	130	46·6	0·018
16–19 years	49	40·5	72	59·5	
Height (cm)					
Mean	150·3		151·1		0·29
SD	8·5*		7·3*		
Weight (kg)					
Mean	40·1		40·7		0·53
SD	9·1		7·7		
BMI (kg/m^2^)					
Mean	14·9		15·1		0·61
SD	2·5		2·2		
Normal	68	48·9	71	51·1	0·97
Thinness (BMI for age <-2 SD)	52	48·6	55	51·4	
Severe thinness (BMI for age <-3 SD)	67	50·0	67	50·0	
Height for age					
Normal	151	49·0	157	51·0	0·97
Stunted (height for age <-2 SD)	30	50·9	29	49·1	
Severe stunting (height for age <-3 SD)	7	50·0	7	50·0	
Religion					
Hindu	182	48·3	195	51·7	0·06
Muslim/Christian/Sikh/Others	14	70·0	6	30·0	
Caste					
Scheduled caste and tribes (SC/ST)	87	43·3	114	57·9	0·003
Other backward classes	21	77·8	6	22·2	
Others	70	47·6	77	52·4	
Family type					
Nuclear family	123	45·7	146	54·3	0·029
Joint family	73	57·5	54	42·5	
Per capita income (Rs.)					
P_50_	1225		1400		0·40
P_25_-P_75_	833–2000†		857–2000†		
Wealth index					
Poorest	40	58·8	28	41·2	0·23
Poorer	35	48·0	38	52·0	
Middle	34	42·0	47	58·0	
Richer	40	46·0	47	54·0	
Richest	49	54·4	41	45·6	
Type of house					
Kutcha	7	58·3	5	41·7	0·49
Semi-pucca	31	55·4	25	44·6	
Pucca	158	48·0	171	52·0	
Name of water source					
Community RO/filter	11	47·8	12	52·2	<0·001
Tubewell	12	22·6	41	77·4	
Piped water	78	54·5	65	45·4	
Handpump	95	53·4	83	46·6	
Frequency of washing hands before consuming food					
Always	169	49·0	176	51·0	0·69
Sometimes	27	51·9	25	48·1	
Agent used for washing hands before consuming food					
Soap	44	46·3	51	53·7	0·50
Only water	152	50·3	150	49·7	
Type of toilet facility at home					
Flush or pour flush toilet	170	48·4	181	51·6	0·30
Pit latrine/use open space or field	26	56·5	20	43·5	
Type of diet‡					
Vegetarian	157	49·4	161	50·6	0·73
Non-vegetarian	15	55·6	12	44·4	
Eggitarian	24	46·1	28	53·9	
Consumption of deworming tablets (past 1 year)					
No	31	43·1	41	56·9	0·23
Yes	166	50·9	160	49·1	
Consumed IFA tablets at school (past 30 d)					
Yes	172	51·8	160	48·2	0·020
No	23	35·9	41	64·1	
Status of menstruation					
Menstruating	123	43·8	158	56·2	<0·001
Not menstruating	75	63·0	44	37·0	

* Values are expressed as Mean ± sd.

† Values are expressed as P_50_ (P_25_–P_75_).

‡ Vegetarian diet includes cereals, pulses, fruits and milk and milk products.

Eggitarian diet includes vegetarian diet + eggs.

Non-vegetarian diet includes eggitarian diet + and meat products.

On adjusted analysis, anaemic participants had significantly lower ferritin (*β*, −0·38; *P* < 0·001), folate (*β*, −1·95; *P* < 0·001) and higher hepcidin levels (*β*, 31·16; *P* = 0·047). However, the serum levels of sTfR, sTfR log ferritin and vitamin B_12_ were comparable in both the groups (Table 3).

**Table 3:** Comparison of anaemia-related biomarkers in the two groups

Parameters	Non-anaemia group			Anaemia group			*P* value	*β* coefficient§	95 % CI	*P* value 2§
	*n*	Mean	95 % CI	*n*	Mean	95 % CI				
Ferritin (log) (ng/ml)*	188	3·3	3·2, 3·4	200	2·8	2·7, 2·9	<0·001	−0·38	−0·55, −0·21	<0·001
Soluble transferrin receptor (mg/l)	168	1·5	1·4, 1·6	147	1·4	1·2, 1·6	0·70‡	−0·04	−0·29, 0·20	0·71
P_50_ (P_25_–P_75_)		1·4	1·1, 1·9		1·6	0·2, 2·0				
Soluble transferrin receptor/log ferritin (mg/μg)†	159	0·5	0·4, 0·5	145	0·5	0·4, 0·6	0·18‡	0·06	−0·04, 0·16	0·25
P_50_ (P_25_–P_75_)		0·4	0·3, 0·6		0·5	0·1, 0·7				
Hepcidin (ng/ml)	182	170·4	149·3, 191·6	180	203·5	182·4, 224·5	0·13‡	31·16	0·46, 61·86	0·047
P_50_ (P_25_–P_75_)		114·8	89·3, 182·5		123·0	86·8, 312·9				
Serum folate (ng/ml)	182	9·7	9·2, 10·2	189	7·7	7·1, 8·3	<0·001‡	−1·95	–2·76, −1·14	<0·001
P_50_ (P_25_–P_75_)		12·0	7·4, 12·0		8·8	3·7, 12·0				
Vitamin B_12_ (pg/ml)	188	245·4	234·7, 256·1	200	250·7	240·6, 260·7	0·48	1·57	−14·12, 17·26	0·84
P_50_ (P_25_–P_75_)		234·0	198·0, 282·5		238·0	202·5, 280·5				

* Ferritin values were log transformed.

† Soluble transferrin receptor/log ferritin ratio estimates the body Fe across normal and depleted Fe stores.

‡ Mann–Whitney test performed.

§ *P* value 2: Adjusted for age, CRP, caste, source of water, reported consumption of IFA and status of menstruation.

### Characterisation of anaemia

Anaemic girls had significantly higher proportion of Fe deficiency as compared with non-anaemic girls (IDA: 29·6 % *v*. Fe deficiency with no anaemia 11·0 %, *P* < 0·001) (Table 4). High prevalence of folate or vitamin B_12_ deficiency was observed in both the anaemic (28·1 %) and non-anaemic groups (29·5 %). However, isolated folate deficiency was higher in anaemic subjects (25·1 % *v*. 8·2%; *P* < 0·001). Mixed deficiency characterised by deficiency of Fe with folate or vitamin B_12_ was higher in anaemic participants (15·8 % *v*. 6·4 %; *P* = 0·004). Acute inflammation was found in 9·7 % anaemic and 14·4 % non-anaemic girls. Anaemia of other causes was observed in 16·8 % anaemic participants.

**Table 4:** Characterisation of anaemia amongst adolescent girls aged 12–19 years

Parameters	Non-anaemia group			Anaemia group			*P* value	Odds ratio*	95 % CI	*P* value 2*
	Total *n*	Deficient %	95 % CI	Total *n*	Deficient %	95 % CI				
Fe deficiency	173	11·0	7·1, 16·6	196	29·6	23·6, 36·4	<0·001	2·49	1·34, 4·62	0·004
Folate or vitamin B_12_ deficiency	173	29·5	23·1, 36·7	196	28·1	22·2, 34·8	0·76	–		–
• Isolated folate (<4 ng/ml)	182	8·2	5·0, 13·2	203	25·1	19·6, 31·5	<0·001	3·95	2·03, 7·71	<0·001
• Isolated vitamin B_12_ (<203 pg/ml)	188	28·7	22·7, 35·6	213	26·8	21·2, 33·1	0·66	–		–
Mixed deficiency	173	6·4	3·5, 11·1	196	15·8	11·3, 21·7	0·004	2·40	1·15, 4·99	0·019
Inflammation	173	14·4	9·9, 20·6	196	9·7	6·2, 14·7	0·16	–		–
Other causes	–	–		196	16·8	12·2, 22·8		–		–

* *P* value 2: Adjusted for age, caste, source of water, CRP, reported consumption of IFA and status of menstruation.

Anaemic participants had significantly higher odds of having Fe deficiency (AOR: 2·49, 95 % CI (1·34, 4·61), *P* = 0·004), isolated folate deficiency (AOR: 3·95, 95 % CI (2·03, 7·71), *P* < 0·001) and mixed deficiency (AOR: 2·40, 95 % CI (1·15, 4·99), *P* = 0·019) (Table 4).

## Discussion

The study population had comparable stunting rates but higher prevalence of thinness, severe thinness than the national average for adolescents in the age group of 10–19 years^(1)^. However, in congruence with an earlier systematic review^(22)^, anaemia had no association with BMI and stunting amongst adolescent girls. Adolescent girls above 15 years had higher proportion of anaemia possibly due to the high Fe demands of the body due to mensuration and for supporting accelerated growth^(23)^. In the present study, half of the adolescent girls belonged to SC/ST and anaemia was found to be highest amongst them. In India, a bulk of this SC/ST population fall in the lowest two wealth brackets, which breeds subclinical infection and relatively poorer quality of diets.

Our study characterises anaemia amongst rural adolescent girls in India. Fe deficiency (29·6 %) was the leading contributor of anaemia followed by folate and vitamin B_12_ deficiency (28·1 %). Mixed deficiency of Fe, folate and vitamin B_12_ was found in 15·9 % of the anaemic girls. Deficiencies of Fe, folic acid, vitamin B_12_ and their mixed deficiency together contributed to 73·5 % of the anaemia amongst adolescent girls. The recent Comprehensive National Nutrition Survey conducted in India amongst 1912 anaemic adolescent girls reported similar proportion of Fe deficiency (26·8 %) and folate or vitamin B_12_ deficiency (22·2 %)^(5)^. Findings of the present study are consistent with the global evidence that Fe deficiency contributes to less than half of the nutritional anaemia amongst Indian adolescent girls^(3,24,25)^. The Biomarkers Reflecting Inflammation and Nutritional Determinants of Anemia project consisting of nationally representative data from ten surveys of women of reproductive age (15–49 years), reported that in low- and middle-income countries where inflammation levels were high, Fe deficiency (7–35 %) played a much smaller role in the etiology of anaemia^(24)^. Another systematic analysis of National surveys found that in countries where anaemia prevalence was >40 %, especially in rural populations, Fe deficiency contributed to only 16 % anaemia among women of reproductive age^(4)^.

In the present study, more than 10 % of the participants were found to have Fe deficiency with no anaemia. Fe deficiency may be present despite a normal haemoglobin and full blood count as Fe deficiency anaemia is a late manifestation of Fe deficiency^(26–28)^. Fe deficiency with no anaemia has been associated with many symptoms such as menorrhagia^(29)^, dizziness^(29)^, dyspnoea^(29)^, fatigue^(30)^, reduced quality of life^(31)^ and mood disturbances^(32)^.

The combined deficiency of folate and vitamin B_12_ deficiency was presented in more than one-fifth of the anaemic and non-anaemic girls. Anemic girls had significantly lower isolated folate levels and folate deficient girls had 4·17 times higher odds of anaemia. Contrary to other studies^(33,34)^, a cross-sectional research conducted amongst Indian tribal adolescent boys and girls aged 10 to 17 years reported no association of anaemia with folate deficiency. They also reported that girls were 3·8 times more likely than boys to be deficient in folate^(35)^. Earlier studies conducted in Kuwait also reported no significant association of folate with anaemia and <10 % prevalence of anaemia in the adolescents with low folate status^(36,37)^.

Folate is known to work closely with vitamin B_12_ during erythropoiesis. Deficiency of folate slows formation and maturation of RBC. Low concentration of vitamin B_12_ in turn contributes to the reduced formation of metabolically active folate, reduced folate retention in developing RBC and indirect induction of intracellular folate deficiency. Thus, coexisting vitamin B_12_ deficiency has been suggested to negatively affect and further precipitate folate deficiency^(38)^.

The role of vitamin B_12_ in the aetiology of anaemia has been inconsistent^(3,39,40)^. In the present study, serum vitamin B_12_ was similar between both anaemic and non-anaemic groups. A study conducted to assess the haematological status of Indian adolescent girls residing in an urban slum in West Delhi reported that serum levels of serum ferritin, folic acid and vitamin B_12_ decreased and the deficiency increased linearly with the increasing severity of anaemia^(34)^. Similar trends in serum ferritin, folic acid and vitamin B_12_ were reported by another cross-sectional hospital-based study among 200 Indian adolescents (10–18 years) with anaemia^(33)^. However, earlier RCT assessing the effect of supplementation of vitamin B_12_ have also shown no beneficial effect on haemoglobin levels^(41,42)^. Studies have suggested that anaemia due to vitamin B_12_ deficiency does not usually appear until an individual has a relatively severe state of depletion^(43,44)^. Estimation of one biomarker of circulating vitamin B_12_ (serum vitamin B_12_ or holotranscobalamin) and one functional biomarker (MMA or tHcy) may specify the true vitamin B_12_ status^(45)^. Hence, further studies may utilise an additional functional biomarker to document its association with anaemia.

In our study, high prevalence of inflammation was observed amongst both anaemic (9·7 %) and non-anaemic (14·4 %) adolescent girls. Anaemia of inflammation deregulates the synthesis of hepcidin, master regulator of Fe homoeostasis. This leads to the degradation of ferroportin-1 exporter and sequestration of Fe and therefore inhibiting the entry of Fe into plasma and systemic circulation causing a pseudo deficiency situation^(46)^ and reduced Fe bioavailability^(47)^. Urinary and serum hepcidin has been documented to increase to up to 100-fold during infections and inflammation. Hepcidin levels may therefore distinguish individuals with Fe deficiency anaemia *v*. anaemia of inflammation^(47)^.

We found that the mean hepcidin levels were higher amongst anaemic adolescent girls. Hepcidin was documented to be significantly associated with haemoglobin, ferritin and sTfR amongst Sri Lankan adolescents^(48)^. A recent study conducted to assess the relative maturity of serum hepcidin in the setting of IDA reported differing results as hepcidin was significantly lower in children (6 months – 18·2 years) with IDA than in the control participants^(49)^. The authors concluded that inhibited maturation and synthesis of hepcidin in children and unadjusted values of hepcidin for age may have resulted in its low concentration in IDA^(49)^.

A possible source of inflammation may have been the water from unclean water sources as more than 40 % of the rural adolescent girls consumed untreated water from the handpumps. Inflammation may have been further exacerbated by poor hand washing practices as majority (75·5 %) of the girls washed their hands only with water before consuming food. Chronic intestinal inflammation can lead to atrophy of the intestinal villi and impaired absorption of haematopoietic nutrients from the diet, without obvious diarrhoea^(50,51)^. Evidence suggests that children exposed to long-term poor quality of water, sanitation and hygiene conditions and open defecation have lower haemoglobin levels due to increased risk of intestinal infection and chronic gut inflammation^(52,53)^.

Our study was conducted in rural areas, and the markers for nutritional anaemia such as Fe deficiency including the utilisation marker hepcidin, folate, vitamin B_12_ deficiency and inflammation were evaluated simultaneously in both anaemic and non-anaemic adolescent girls. However, the following limitations merit consideration. We utilised filter paper for estimation of haemoglobin due to logistic reasons. This could have underestimated haemoglobin and overestimated anaemia^(54)^. Further, budgetary constraints precluded evaluation of other causes of anaemia including haemoglobinopathies, vitamin A status and hookworm infestation and chronic inflammation using *α*-1-acid glycoprotein. Adolescent girls who were not going to school could not be included due to feasibility issues. Another limitation of the study is that we did not collect information on the history of diarrhaeal episodes.

Prevention and control of anaemia in adolescent girls, the future mothers, needs to be addressed on a priority. The current strategy of IFA supplementation alone will not suffice for achieving the desired reduction in the prevalence of anaemia as unknown causes (16·8 %), and anaemia of inflammation (9·7 %) contributes to a substantial proportion of anaemia.

In addition, high prevalence of Fe, folic acid, vitamin B_12_ deficiencies and inflammation among non-anaemic girls places them at an increased risk of developing anaemia. Currently, no government strategy exists for testing and addressing these deficiencies in non-anaemic children, even amongst those presenting with symptoms.

The findings of our study suggest development and implementation of strategies that converge anaemia-specific programme with all other nutrition-specific components like improving water, sanitation and hygiene practices. Such strategies will comprehensively tackle anaemia by reaching all anaemic and non-anaemic adolescent girls. Further studies are needed to characterise the additional important determinants of anaemia in adolescents.

### Highlights of the study

1. Fe deficiency was the leading contributor of anaemia amongst rural adolescent girls followed by folate deficiency. The deficiency of these micronutrients along with their mixed deficiency contributes to 73.5 % of the anaemia.
2. Anaemia of inflammation (9.7 %) contributes to a substantial proportion of anaemia.
3. High prevalence of Fe, folic acid, vitamin B_12_ deficiencies and inflammation also exists among non-anaemic groups.
4. Unknown causes of anaemia amongst one-sixth of the anaemic adolescent girls warrant the need for further studies to understand the aetiology of anaemia.
