# Characterisation of anaemia amongst school going adolescent girls in rural Haryana, India – CORRIGENDUM

**DOI:** 10.1017/S1368980023001465

**Published:** 2023-10

**Authors:** Aakriti Gupta, Harshpal Singh Sachdev, Umesh Kapil, Shyam Prakash, Ravindra Mohan Pandey, Hem Chandra Sati, Lokesh Kumar Sharma, Priti Rishi Lal

In the published article, the authors overlooked mentioning that 0.5g/dl was added to the original hemoglobin values to correct for the difference using direct and indirect cyanmethaemoglobin method.

The authors apologise for this error and state that this does not change the scientific conclusions of the article in any way.

## References

[ref1] Gupta, A. , Sachdev, H. , Kapil, U. , Prakash, S. , Pandey, R. , Sati, H. , … Lal, P. (2022). Characterisation of anaemia amongst school going adolescent girls in rural Haryana, India. Public Health Nutrition, 25(12), 3499–3508. doi: 10.1017/S1368980022000210 PMC9991616 35067260

